# Polymorphic Expression of UDP-Glucuronosyltransferase UGTlA Gene in Human Colorectal Cancer

**DOI:** 10.1371/journal.pone.0057045

**Published:** 2013-02-27

**Authors:** Min Wang, De-Feng Sun, Shuai Wang, Ying Qing, Shuo Chen, Dong Wu, Ying-Min Lin, Ji-Zhuang Luo, Yan-Qing Li

**Affiliations:** 1 Department of Geriatrics and Gastroenterology, Qi-Lu Hospital of Shandong University, Key Laboratory of Proteomics of Shandong Province, Jinan, Shandong Province, China; 2 Department of Biochemistry and Molecular Biology, Shandong Medical School, Jinan, Shandong Province, China; 3 Department of General Surgery, Qi-Lu Hospital of Shandong University, Jinan, Shandong Province, China; 4 School of life science, Shandong University, Jinan, Shandong Province, China; 5 Department of Gastroenterology, Qi-Lu Hospital of Shandong University, Jinan, Shandong Province, China; Sapporo Medical University, Japan

## Abstract

**Background:**

Polymorphism of genes encoding drug-metabolizing enzymes is known to play an important role in increased susceptibility of colorectal cancer. UGT1A gene locus has been suggested to define tissue-specific glucuronidation activity. Reduced capacity of glucuronidation is correlated with the development of colorectal cancer. Therefore, we sought to explore polymorphism of UGTlA gene in human colorectal cancer.

**Methods:**

Cancerous and healthy tissues were obtained from selectedpatients. Blood samples were collected and UGTlA mRNA transcriptions were analyzed. Genomic DNA was prepared and UGTlA8 exon-1 sequences were amplified, visualized and purified. The extracted DNA was subcloned and sequenced. Two-tailed Fisher's exact test, Odds ratios (ORs), confidence interval (CIs) and Logistics Regression Analysis were used for statistical analysis.

**Results:**

UGTlA mRNA expression was reduced in cancerous tissues compared with healthy tissues from the same patient . The UGTlA mRNA expression of healthy tissue in study patients was lower than control . The mRNA expression of cancerous tissue was down-regulated in UGTlAl, 1A3, 1A4, lA6, 1A9 and up-regulated in UGTlA8 and UGTlAl0 UGT1A5 and UGT1A7 were not expressed in colonic tissue of either group. The allele frequency of WT UGTlA8*1 was higher (p = 0.000), frequency of UGTlA8*3 was lowered in control group (p = 0.000). The expression of homozygous UGTlA8*1 was higher in control group (p = 0.000). Higher frequency of both heterozygous UGTlA8*1/*3 and UGTlA8*2/*3 were found in study group (p = 0.000; p = 0.000). The occurrence of colorectal cancer was mainly related to the presence of polymorphic UGTlA8*3 alleles (p = 0.000).

**Conclusion:**

Regulation of human UGT1A genes is tissue-specific. Individual variation in polymorphic expressions of UGTlA gene locus was noted in all types of colonic tissue tested, whereas hepatic tissue expression was uniform. The high incidence of UGTlA8 polymorphism exists in colorectal cancer patients. UGTlA8*1 allele is a protective factor and UGTlA8*3 allele is a risk factor.

## Introduction

Colorectal cancer is one of the most common types of cancer worldwide. Based on data from International Agency for Research on Cancer (IARC), colorectal cancer accounts for 9.4% of all *de novo* cancers diagnosis in 2007.Compare to the figures in 2000, newly diagnosed colorectal cancer in 2007 had increased by 27%, and mortality rate had risen by 28%; which accounts for an annual increase of 3.9% and 4.0%, respectively[1].

Although the etiology of colorectal cancer is not fully understood, Polymorphism of genes encoding drug-metabolizing enzymes is known to play an important role in increased susceptibility of colon cancer[2]. A myriad of enzymes are involved in phase I and phase II metabolic pathways, including members of the cytochrome P450 (P450) and UDP-glucuronosyltransferase (UGT) superfamilies. UGTs catalyze glucuronidation reaction, which is important for xenobiotic metabolism. Glucuronidation allows for the utilization of certain nutrients as well as detoxification and excretion of potentially harmful compounds and metabolites, such as steroids, bilirubins, exogenous drugs, toxic chemicals and mutagenic substances[3]. Over 50 types UGTs had been identified in many organs and tissues of both animals and human, including liver, intestinal mucosa, lungs, brain, placenta and uterus [4]–[8]. Human UGTs have been divided into the UGT1, 2, 3 and 8 multigene families based on evolutionary divergence[4]. To date, 21 different UGTs[9] have been found in humans. The human UGT1A gene locus is located on chromosome II. It is composed of thirteen different first exons on the 5′ end linked, by alternative splicing, to four different common exons on the 3′ end. UGT1A gene locus encodes at least nine functional proteins (UGT1A1, UGT1A3-UGT1A10) and four pseudogenes (UGT1A2, UGT1A11-UGT1A13)[10].

Tissue-specific expression of the UGT1A gene locus has been well characterized. The gene had been suggested to define tissue-specific glucuronidation activity in human digestive system. Hepatic UGT1A proteins (UGT1A1, UGT1A3, UGT1A4, UGT1A6, and UGT1A9) have been studied and identified in detail. Studies examining human gastrointestinal tract have led to the identification of three extrahepatic UGT1A transcripts: UGT1A7, UGT1A8 and UGT1A10[11], [12]. Using Reverse Transcriptase Polymerase Chain Reaction (RT-PCR), lower gene expression of UGT1A8 was noted in the esophagus [13]. A more comprehensive study showed no detectable UGT1A8 RNA in the small intestine but abundant levels of UGT1A8 mRNA in the large intestine[12] . Presently, data on the expression of UGT1A8 in liver or any other human tissue has been scarce. The selective expression of UGT1A8 in colon could indicate that UGT1A8 plays an important role in cellular homeostasis and the disposition of endogenous and exogenous compounds. It was reported that human UGT1A8 protein catalyze glucuronidation of coumarins, phenolic compounds, anthraquinones, flavonoids and a number of steroids, on transfected human embryonic kidney 293 (HEK293) cells [14]. Consider the fact that colon contains a considerable amount of UGT1A proteins[12] , a reduced capacity of glucuronidating specific substances could be detrimental to the homeostatic balance of the colon.

During the past years, genotyping and sequencing data had led to the discovery of over 100 variants within the promoter regions and the coding sequence of the UGT1A genes. Notably, many of the variants exhibit allele frequencies of up to 40–50% in the general population, which are found to be in linkage disequilibrium. But, a few variants are of sufficient frequency in the general population to be classified as polymorphisms[15], [16]. Polymorphism is best represented by the UGT1A1 gene, which is known to contain 113 variant allele genotypes of UGT1A1 (UGT1A1*1-UGT1A1*113). Many of UGT1A1 genes exhibit high allele frequencies[17], [18]. Genetic polymorphism had also been found in UGT1A3[19], [20] , UGT1A4[21], [22] , UGT1A6 [23], [24], UGT1A7[16], [25], [26], UGT1A8 [12], [14], [21], [27], [28], and UGT1A9[29] . Polymorphism leads to different degrees of transcriptional as well as functional alterations, which may decrease UGTs activity and results in pathology of the affected individuals. The analysis of a case-controlled study revealed increased risk of developing colorectal cancer (CRC) in individuals carrying UGT1A1*6 and UGT1A7*3 variants[30] . The analysis of genetic polymorphisms of UGT1A3 genes (UGT1A3*2, UGT1A3*3) showed that not only the activity level of the expressed UGT1A3 protein had changed but the specificity for different substrates was affected as well[20]. Extensive studies had been performed on UGT1A7 gene variants. With its low carcinogen metabolizing activity, UGT1A7 gene is a risk factor for hepatocellular carcinoma (HCC) development in, Chinese (OR = 3.06)[31], French (OR = 3.4)[32], Japanese (OR  = 2.33)[33] , and Koreans (OR  =  1.45)[34]. In addition, UGT1A4*2 and UGT1A4*3 was also considered as a risk factor for HCC in an allelic association study[21] .

The catalytic efficiency of UGT1A8*3 (C277Y) and UGT1A9*3 (M33T) allozyme toward mycophenolic acid was drastically decreased[29]. Allele variation in one of the many UGT loci can leads to significant biochemical alterations in drug metabolizing potential. Those UGT enzymes are known to degrade carcinogens; a lack of function may play an important role in the etiology of a carcinogenic episode such as colorectal cancer.

To achieve a better understanding of the role of UGT1A in the gastrointestinal tract, the current study had focused on UGT1A8 in the gastrointestinal tract. Based on earlier reports[35], we recognized changes of expression and function of UGT1A8 might be a risk factor of colorectal cancer. In this study, polymorphic expression of UGT1A genes was examined. We tested genotype of UGTlA8 in patients with colorectal cancer and explored relationship between polymorphism of UGTlA8 genes and colorectal cancer.

## Materials and Methods

### 2.1 Clinical material

(1)In colorectal cancer group, 150 patients (90 women and 60 men, mean age 56.8±11.3 years) were screened in our General Surgery Department, Qilu Hospital, Shandong University, Shandong province, China. These patients were selected according to standard of Amsterdam II[36] , precluding hereditary nonpolyposis colorectal cancer (HNPCC) and familial adenomatous polyposis (FAP). Eligibility was determined if primary diagnosis occurred up to six months prior to study enrollment, with final diagnosis confirmed through Pathology Department.

None of the patients received chemotherapy, radiotherapy or biotherapy prior to samples collection. 120 normal subjects (48 women and 72 men, mean age 57.2±11.9 years) were screened from Department of Interventional Gastroenterology in Qilu Hospital, Shandong University. 72 normal hepatic tissue samples from volunteers (30 women and 42 men, mean age 56.5±10.2 years) were obtained through Department of General Surgery, Qilu Hospital of Shandong University. Serial markers of serum hepatitis virus were negative in these 72 subjects. Ten subjects were referred for hepatic haemangioma and fourteen were referred for hepatic cyst. Review of medical records in all aforementioned subjects indicated the absence of chronic drug taking behaviors, as well as the absence of smoking and alcohol abuse. Additional sample normalization measures were taken, including smoking cessation 6 months prior to tissue sample collection, and fasting at least 12 hours prior to the surgical procedures and tissue collection.

(2) 327 patients (123 women and 204 men, mean age 56.4±11.0 years) with colorectal cancer were screened in Department of General Surgery and Department of Gastroenterology, Qilu Hospital,Shandong University. These patients were selected based on standards of Amsterdam II criteria as before[36]. In control group, there were 327 gender-matched volunteers (average age 54.2±10.3 years). On average, volunteers were two years younger than colorectal cancer patients (p < 0.05). All subjects were Han people coming from the common area of Shandong province in China, without consanguinous relationship. Questionnaires were administered by specially trained registered nurses to study subjects in-person. The questionnaire collected information on lifestyle factors such as physical activity; alcohol and tobacco usage; medical, family, and work history; and the use of over-the-counter medications. Additionally, in order to preserve epidemiological archives and assess individual exposure to dietary carcinogens, detailed questionnaire was used to measure average dietary intake one year before diagnosis of colorectal cancer, or one year before the date of selection for controls. There were no other significant differences (e.g., by age, family history of colorectal cancer, smoking status, total meat intake, education level or income) between individuals who provided blood sample and general population. Informed Consent was obtained, and the study was approved by the ethics committee of Shandong University.

### 2.2 Tissue samples and reagents

(1) In colorectal cancer group, 150 pairs of samples (1 cm×1 cm×1 cm) were harvested by operating surgeons from 150 patients with colorectal cancer during partial colectomy. Samples were then chilled in 0°C saline. Each pairs of sample consisted of malignant tissue and surrounding healthy tissue. Healthy tissue was harvested at a distance of more than 5 cm from resection margin of colorectal cancer. 120 healthy colon mucosal samples from control group were harvested through electrical enteroscope. Macroscopic examination of healthy mucosa from study group and control subjects showed no signs of deterioration such as necrosis. Microscopic examination with light microscope documented normal histology. The tissues were free of tumor and any detectable concurrent disease such as colitis, dysplasia. Colonic mucosa was dissected and to obtain tissue samples free of colon muscularis and most of the submucosa. This allowed for a direct comparison with hepatic epithelium by minimizing the presence of nonepithelial cell types. 72 hepatic tissue samples of volunteers were obtained through laparoscope at a distance of more than 5 cm from the edge of surgical ablation. Samples were selected for the absence of histologically apparent disease such as hepatitis, and liver cirrhosis to minimize sample bias. After being encapsulated in tin foil wrapper and labeled, all tissue samples were rinsed in cold 0.9% NaCl, and immediately frozen in liquid nitrogen within 10 minutes of surgical removal and were continuously stored at −80°C until further use.

Reverse transcriptase MMLV was purchased from Sigma Chemical Co. TaqDNA polymerase was purchased from Shanghai Shengong Co. Reagents needed in reaction systems of Reverse Transcription (RT) and Polymerase Chain Reaction (PCR) were provided by Tumor Immunityand Genetic Engineering Key Laboratories. UGTlA kit was purchased from Gentest Corp.

(2) 5 ml of peripheral venous whole blood specimens was collected from 327 of colorectal cancer patients and 327 cases from healthy adults. Both groups were fasted at least 12 hours prior to sample collection. Acid citrate dextrose (ACD) was used to prevent blood coagulate until analysis. Reagents of erythrocyte lysate, leucocyte lysate, protein precipitation and DNA hydration were provided from Medical Genetics Institute of Medical College in Shandong University. Reagents needed in reaction systems of RT-PCR and TaqDNA endonuclease were purchased from Promega CO. PCR products gelatin retrieving kit was purchased from BioTek CO. All other reagents were obtained from commercial sources and of analytical grade.

### 2.3 Detection of UGTlA mRNA expression in different tissues

The presence of UGT1A transcripts in total tissue RNA was analyzed by PCR amplification, performed as a duplex RT-PCR co-amplification with β-actin cDNA as a control. RT-PCR detection of all UGT1A transcripts predicted by the human UGT1A locus was performed using exon 1 specific sense primers and antisense primers, located within exons 2–5 or within a common portion of the 3'end of the first exons. All primers were biosynthesized from Shenggong CO., Shanghai, after retrieving in genomic laboratory, as shown in Table S1.

RNA isolation was prepared according to Guanidinium Isothiocyanate methods. Approximately 200 mg of frozen tissue was pulverized in a mortar filled with liquid nitrogen. Tissue powder was immediately lysed in acidic phenol-guanidinium isothiocyanate solution. UGT1A cDNA was co-synthesizd with β-actin cDNA in Eppendorf tubes containing 1 µg RNA, 1 µl MMLV, 7 µl RT-PCR system, 1 µ1 downstream primers .UGT1A cDNA was co-amplified with β-actin cDNA in a starting volume of 98 µl containing 20 µl reverse transcription products, 19 µl PCR system, 1 µl upstream primers, 58 µl ultra-pure water. After incubation at 94°C for 3 min, PCR cycle was started by adding 2 µl TaqDNA polymerase followed by centrifugating. A total of thirty-five cycles was performed. Each PCR cycle consists of amplification reactions at 94 for 1 min, 58°C for 1 min, and 72°C for 1 min, followed by elongation reactions with extended elongation time of 7 min at 72°C. Analytical agarose gel electrophoresis was performed to identify PCR products. Kodak Gelatin Analysis System was used to evaluate expression intensity of UGT1A and UGT1A isoforms according to the following formula: 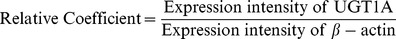



Experiments were performed with multiple levels of controls, including controls without cDNA, primers, or thermophilic polymerase. Specificity of this assay was determined by PCR using all primer pairs on each cloned template cDNA to exclude cross-reactivity. To confirm the detection of specific UGT1A cDNA using this assay, the PCR products were partially sequenced to document the identity of these specific gene products.

### 2.4 DNA isolation from whole blood samples

300 µl of blood specimens were infused into 1.5 mL Eppendorf tubes. 900 µl of erythrocyte lysate was added to the sample. Digestion was allowed for 10 minutes at room temperature. The sample was then centrifuged at 12,000 g for 20 seconds and the clear supernatants were removed. 50 µl of erythrocyte lysate and 300 µl of leukocyte lysate was added to resuspend the pellet, followed by 30 minutes of digestion at room temperature. Protein was precipitated by adding 100 µl of protein precipitation and the sample was then centrifuged at 12,000 g for 20 seconds. The supernatant were transferred to a new 1.5 ml Eppendorf tube, followed by slow instillation with double-volumed pre-chilled dehydrated ethanol. The sample was vacillated slightly until DNA precipitation. The sample was then centrifugation at 9,000 g for 1 minute and the clear supernatants were removed. 500 µl of 75% ethanol was added to the precipitate, followed by centrifugation at 9,000 g for 1 minute, supernatants were removed and DNA precipitate was allowed to air dry. DNA hydration was added to rehydrate DNA and dilute DNA solution to a concentration of 20 mg/L for further use, after quantification of DNA by ultraviolet spectrophotometer. DNA was rehydrated with pure water to a concentration of 20 mg/L. The concentration was verified with ultraviolet spectrophotometer prior to further use.

### 2.5 Amplification of UGT1A8 exon-1 by PCR

Exon-1, located at the 5'-end of human UGT1A8, presents individual variation in its regulation. The primers were designed based on array of UGT1A gene locus (accession number AF297093). Forward primer (prlA8F, bases34175—34198): 5′-TGG GGT CAG GTT TTG TGC CTG TAG-3′, reverse primer (prlA8F, bases 35175—35208): 5′-GAA ATT GTC AAA TCA CAA TTC AGT AAG GAA TCT -3′. The reaction condition was adjusted to 4 µL of 5×PCR reaction, 4 µL of 5×dNTP, 0.5 µL of forward primer, 0.5 µL of reverse primer, 5 µL of Taq DNA endonuclease, 2 µL of DNA template, and supplemental optical volume of sterile thrice distilled water, which made up a total volume of 100 µL. 35 cycles of amplification reaction was performed. Each cycle consisted of denaturation at 95°C for 30 seconds, annealing reaction at 57°C for 30 seconds, and extension at 72°C for 30 seconds. The entire cycle was preceded by a 3minutes incubation of the reaction mixture at 95°C and followed by 10 minutes elongation at 72°C. Identity of the PCR products was confirmed with gel electrophrosis.

### 2.6 Purication and sequencing of PCR products

PCR products were stained with ethidium bromide for 15 min, and isolated with 20 g/L agarose gel, at a voltage of 120 V for 1 hour. The amplified DNA straps were cut out under ultraviolet lamp at 320 nm and placed into 1.5 mL Eppendorf tubes. Quadrupled volume of Binding Buffer was added to the DNA straps. After incubation with water bath at 65°C for 7 min, the mixture was transferred into columns and centrifuged at 12,000 g for 1 minute. 300 µl of Binding Buffer was used to clean the column after discarding the solution. Another 750 µl of DNA Wash Buffer was added to the sample and centrifugation was repeated at 10,000 g for 1 minute. The mixed solution was centrifuged at 12,000 g for 1 minute prior to discarding the supernatants. After removal of supernatant, samples were transferred from 1.5 ml Eppendorf tubes into column. Then, 30 pl of DNA Water Buffer was added into column, the mixture was then centrifuged at 12,000 g for 1 minute. The solution was collected for quantification and further analysis. The collected DNA fragments of different genotypes were transferred into TOPO TA plasmid The DNA samples were sequenced in Medical Genetics Institute of medical college of Shandong University with a fully automatic sequencer (Prism 377, ABI CO., USA) according to the manufacturer's instructions.

This study used the same quality control measures described by Lesley M. Butler, et al.[37]. First, positive and negative controls were included in each PCR and Taqman experiment. Homozygote wild-type, heterozygote, and homozygote variants for each UGT1A8 genotype from genomic DNA samples of UGT1A8 genotype were included. Second, repeated assays were conducted on three randomly selected samples from each experiment and there was 100% agreement. Third, three additional randomly selected samples were confirmed by direct DNA sequencing. Finally, laboratory personnel were blinded to the case status of the samples.

### 2.7 Statistical analysis

Software package of SPSS11.0 was used to analyze data in this study. Quantitative data are expressed as means ± the standard deviation (χ±s). Comparisons between means of two groups of samples were made with Student’s *t*-test, while multiple group means were analyzed by ANOVA. Results of case-control studies were analyzed using two-tailed Fisher's exact test to determine the distribution difference of multiple alleles between cases and controls. Adjusted ORs and 95% CIs for colorectal cancer were calculated using logistic regression models. CIs of 95% were used unless otherwise stated. Adjusted Ors and 95% CIs were used to evaluate the effects of UGT1A8 alleles, genotype, and gender on susceptibility in colorectal cancer. A p-value of 0.05 or less was defined as statistically significant.

## Results

### 3.1 Variation of expression of UGT lA mRNA

All human UGT1A gene transcripts display a unique 5′ terminus characteristic of each of the individual transferase and a common 3′ portion encoded by exons 2–5. The 3′ region is identical in all members encoded by the UGT1A locus and can therefore be exploited to analyze overall UGT1A expression. Co-amplification of the 487-bp UGT1A and 317-bp human β-actin PCR products, separated in a 2% agarose gel and stained with ethidium bromide, is demonstrated using cancerous tissues, its surrounding healthy tissues, normal colonic tissues from control patients, and hepatic tissues. Breadth and intensity of β-actin (317-bp) in panels were consistent as expected. The breadth and intensity of UGT1A mRNAs (487-bp) were much more variable. Quantification of DRT-PCR products was achieved by calculating relative coefficient using Kodak Gelatin Analysis System. However, the quantification revealed significant differences in steady state levels among three extrahepatic source and hepatic tissues.

In colorectal cancer patients, UGTlA mRNA expression were significantly reduced in pathological tissues compared with the surrounding healthy tissues harvested together . UGT1A mRNA expression in healthy tissue harvested from colorectal cancer patients was significantly lower than healthy colonic tissue harvested from normal control . The highest level of UGT1A mRNA expression was detected in hepatic tissue, which was significantly higher than normal colonic tissues from healthy controls (P<0.01), (Table.1). Colonic UGTlA gene expressions were characterized by significant individual variation, whereas hepatic UGTlA mRNA expressions were more uniform.

**Table 1:** Polymorphic expression of UGTlA mRNA in human tissues.

Tissue samples	cases	relative coefficient of UGT1AmRNA impression
colon cancer tissue	150	0.255±0.137
surrounding healthy tissue	150	0.706±0.235*
normal colonic tissue	140	0.920±0.108†‡
hepatic tissues tissue	72	1.316±0.109§

* P<0.01, †P<0.01 vs. colon cancer tissue; ‡P<0.01 vs. surrounding healthy tissue; §P<0.01 vs. normal colonic tissue.

### 3.2 Polymorphic expression of UGT1A isoforms in different tissues

**Figure 1:**
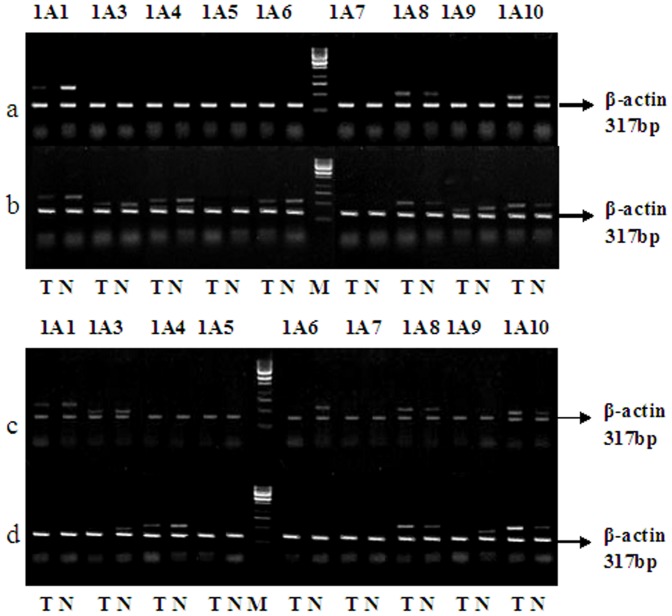
Polymorphic expression of UGTlA mRNA in human tissues. A.T:Colonic cancer tissues; N:Surrounding healthy mucosa; M:Marker

**Figure 2:**
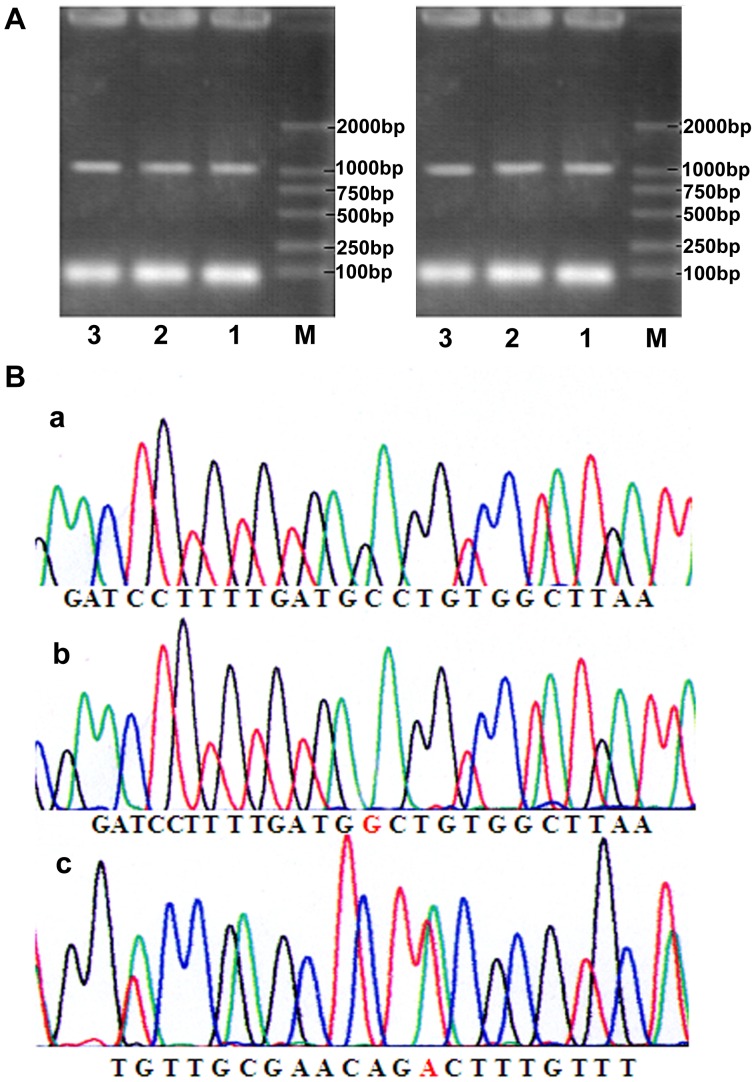
Expression of UGT1AmRNA in whole blood specimens. A.PCR products of UGTlA8 exon-1 (1 033 bp). 1,2,3:PCR products of UGTlA8; M:Marker. B.Sequenced results of uGTlA8 alleles. a:UGTlA8*1;b:UGTlA8*2; c:UGTlA8*3.

Analysis of UGT1A gene expression was performed at the mRNA level employing UGT1A exon 1-specific DRT-PCR. As reported previously, human hepatic and colonic tissues are characterized by a unique and tissue-specific differential expression of the UGT1A locus[11], [12]. Products of exon 1-specific DRT-PCR specifically detects transcripts of all known UGT1A isoforms. DNA straps were separated in 2% agarose and stained with ethidium bromide (Fig.1). Of the UGT1A family isoforms analyzed, UGT1A5 and 1A7 mRNA was not detected in all tissue samples, and the number of samples expressed UGT1A isoforms was different in different tissues. Analysis of 150 different UGT1A transcripts of cancerous tissues demonstrated the following: UGT1A1, 1A8 and 1A10 mRNA were expressed in 102 samples (Fig.1.a). UGTlAl, 1A3, 1A4, lA6, 1A8, 1A9 and 1A10 mRNA were expressed in 54 samples (Fig.1.b). UGTlAl, 1A3, 1A6, 1A8 and 1A10 mRNA were expressed in 66 samples (Fig.1.c). UGTlA3, 1A4, 1A8, 1A9 and 1A10 mRNA were expressed in 108 samples (Fig.1.d). The polymorphic expression of UGTlA isoforms in different tissues was shown in table.2 and fig.2. In contrast to cancerous tissues, UGTlA8 and UGTlAl0 mRNA were not expressed in the 72 different hepatic tissue samples. There was little variation in the abundance of UGTlA isoforms transcripts when each was compared with the β-actin expression levels. UGTlAl, 1A3, 1A4, lA6 and 1A9 mRNA levels were differentially down-regulated in the cancerous tissues compared to surrounding normal mucosa (P<0.01), however, UGTlA8 and UGTlAl0 mRNA levels were up-regulated (P<0.01).

These data demonstrate that the UGT1A locus is expressed in the human gastrointestinal tract. The data also note polymorphic regulation and individual variation of UGTl A gene locus at the RNA transcript. In addition, the identification of tissue-specific expression of UGT1A8 implies that the regulatory mechanisms may be indicative of the evolutionary development and the biological basis determining individual variations in cancer risk.

**Table 2:** The Sample size of UGTlA mRNA polymorphic expression in different tissues.

UGTisoforms	CRC patients (n = 150)			Healthycolorectal mucosa (n = 120)	Healthy hepatic tissues(n = 72)		
	Canceroustissues	The surroundingnormal mucosa					
UGT1A1	102	93		66		72	
UGT1A3	90	69		54		72	
UGT1A4	66	42		42		72	
UGT1A5	0	0		0		0	
UGT1A6	54	33		54		72	
UGT1A7	0	0		0		0	
UGT1A8	150	123		114		0	
UGT1A9	66	60		84		72	
UGT1A10	138	120		108		0	

### 3.3 Identification of array of DNA extracted from whole blood specimens

SeqMan of DNASTAR Software for Molecular Biology was used to analyze the sequences of PCR products. Detected sequences were compared with pre-specified standardized criteria by human genome project [HGP] to determine sites of SNP and frequency of alleles, after pooled analysis of sequenced results and various exclusions of ghost peaks and bad sectors. Population distribution of alleles met Hardy-Weinberg equilibrium by χ2 test (degrees of freedom =  number of alleles-1). The length of PCR products was 1,033bp (Fig.2.A). Sequenced results of purified PCR products indicated that there were three SNPs at base pair 518(CG), base pair 765(AG), and base pair 830(GA) within the coding region of UGT1A8 exon-1(Fig.2.B,Table S2).The mutation at nucleotide 518 leads to missense mutation at codon 173, as a result, alanine is replaced with glycine. The mutation at nucleotide 830 results in substitution of tyrosine for cysteine encoded by codon 277. The mutation at nucleotide 765 is silent mutation. The functional polymorphisms are shown in Table S2. For the majority of the samples with a heterozygous mutation, only a single mutation was present, indicates the three point mutations are not linked (Table S2,S3). To improve accuracy, the collected DNA fragments of different genotypes were transferred into TOPO TA cloning plasmids. Multiple clones from each DNA sample were characterized by DNA sequence analysis. In all cases, the mutation patterns matched the identity of the alleles as outlined in Table S2 and Table.S3. Genotype of samples can be verified using TOPO cloning of DNA fragments, in this case UGTlA8*2 and UGTlA8*3 alleles were identified using TOPO cloning.

### 3.4 Analysis of frequency of alleles and genotype of UGT1A8

**Figure 3:**
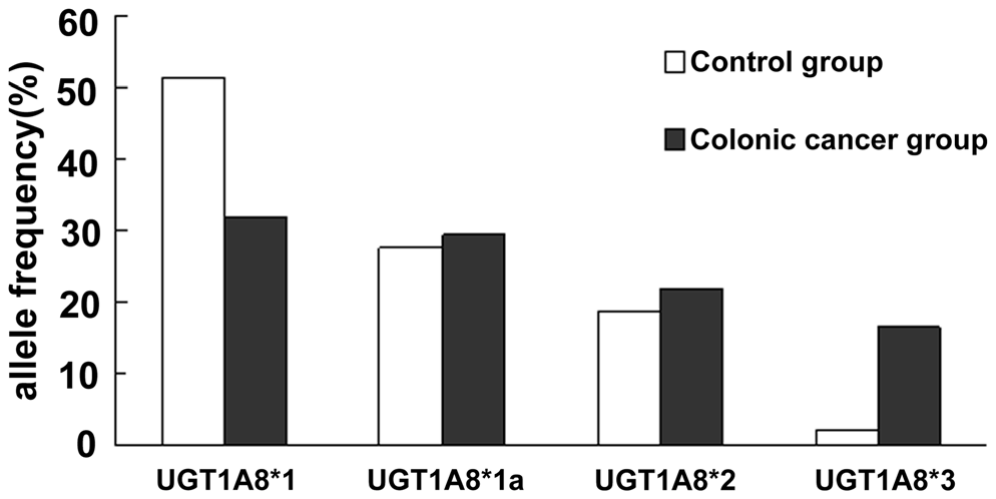
Frequency distribution of UGTlA8 alleles gene (colonic cancer group VS. controls).

**Figure 4:**
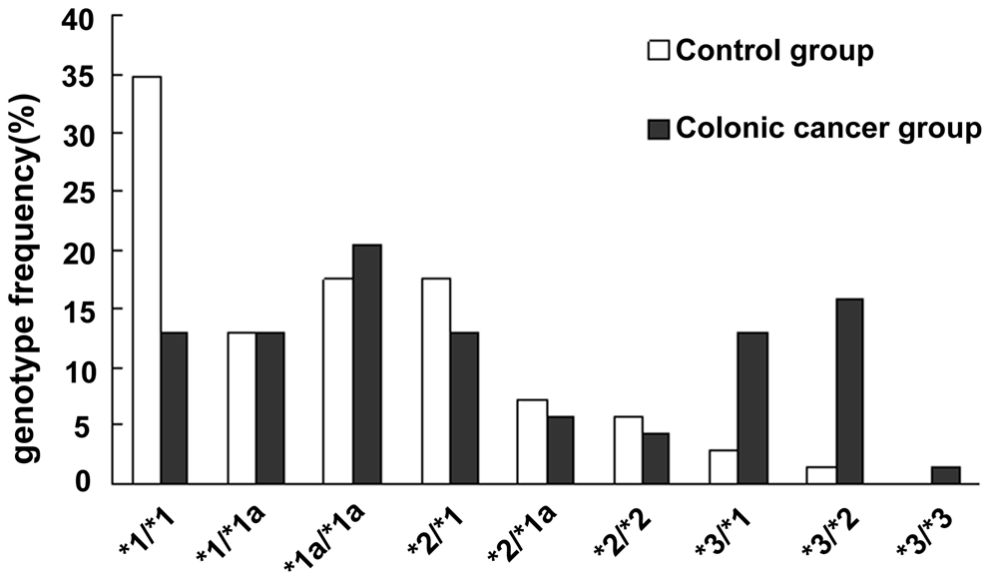
Frequency distribution of UGTlA8 genotype (colonic cancer group VS. controls).

In control group, the allele frequency of UGTlA8*1 (wild type) and UGTlA8*1a was dominant (79%), followed by 18.8% for UGTlA8*2. The identity of UGTlA8*3 was indeed rare, with only 2.3% of the controls observed to carry this genotype (Table.3, Figure.3). UGTlA8*la is a silent mutation and encodes UGTlA8*l. A homozygous pattern of UGTlA8*1 (UGTlA8*1/*1, UGTlA8*1/*1a, UGTIA8*1a/*1a) accounted for about 65.21% of normal adults (Table.4). It was found that the genotype of UGTlA8*1/*2 and UGTlA8*1a/*2 was at a frequency of 24.64%, genotype UGTlA8*1/*3 and UGTlA8*2/*3 was at a frequency of 4.35%, UGTlA8*2/*2 was at a frequency of 5.8% in controls. Interestingly, there was no homozygous UGTlA8*3. In case of patients with colorectal cancer, the allele frequency of UGTlA8*1 and UGTlA8*1a, UGTlA8*2, UGTlA8*3 constituted of approximately 61.5%, 22.0%, and 16.5%(Figure.3), respectively. The heterozygous expression of UGTlA8*1/UGTlA8*2, consist of those with a genotype of UGTlA8*1/UGTlA8*2 and UGTlA8*1a/*2, were at a frequency of approximately 18.84%. 28.98% of individuals with colorectal cancer expressed UGTlA8*1/*3 and UGTlA8*2/*3. The homozygous genotype of UGT1A8 (UGTlA8*1/UGTlA8*1, UGTlA8*2/UGTlA8*2) made up of 46.38%, 4.35% of cancerous cases, respectively. One case of homozygous UGT1A8*3 also noted (Table.3). There were statistically significant differences in allele frequency and genotypes between study group and control group. The wild type allele (UGTlA8*1) frequency was lower among study group than control group [P = 0.000, OR = 0.45, CI (0.28–0.79)]. The same significance could be noted with genotype UGTlA8*1/*l [P = 0.000, OR = 0.28, CI (0.19–0.41)] and UGTlA8*2/*3[P = 0.000, OR = 10.58, CI (4.48–24.98)] (Table.4 and Fig.4 **)**. The opposite results were noted in allele UGTlA8*3 [P = 0.000, OR = 6.99, CI (3.39–14.42)] (Table.3 and Fig.4) and genotype UGTlA8*1/*3[P = 0.000, OR = 5.21, CI (2.49–10.89)]. A conclusion can be drawn from the results of Logistic regression analysis: independent of gender, polymorphism of UGTlA8 is a major risk factor in the development of colorectal cancer (P = 0.000). In particular, UGT1A8*3 allele has the strongest influence (P = 0.000).

**Table 3:** Frequency distribution of UGTlA8 alleles gene (colonic cancer group VS. controls).

Alleles	Frequency of the controls(n = 327)	Frequency of colonic cancer group (n = 327)	P value	F value	OR (95% CI)
UGT1A8*1	0.514(n = 168)	0.321(n = 105)	0.000	25.866	0.448 (0.326–0.615)
UGT1A8*1a	0.275(n = 90)	0.294(n = 96)	0.603		
UGT1A8*2	0.188(n = 60)	0.220(n = 72)	0.242		
UGT1A8*3	0.023(n = 9)	0.165(n = 54)	0.000	37.500	6.989(3.388–14.415)

**Table 4:** Frequency distribution of UGTlA8 genotype. (colonic cancer group VS. controls).

UGT1A8 Genotypes	The control group n = 327(%)	Colonic cancerGroup n = 327(%)	P value	F value	OR (95% CI)
UGT1A8*1/*1	114(34.86)	42(12.84)	0.000	46.618	0.275(0.185–0.409)
UGT1A8*1/*1a	42(12.84)	42(12.84)	1.000		
UGT1A8*1a/*1a	57(17.43)	66(20.18)	0.368		
UGT1A8*2/*1	57(17.43)	42(12.84)	0.102		
UGT1A8*2/*1a	24(7.33)	18(5.50)	0.339		
UGT1A8*2/*2	18(5.50)	15(4.59)	0.592		
UGT1A8*3/*1	9(2.75)	42(12.84)	0.000	23.936	5.207(2.491–10.886)
UGT1A8*3/*2	6(1.83)	54(16.51)	0.000	45.063	10.582(4.484–24.976)
UGT1A8*3/*3	0(0)	6(1.83)	–		–

## Discussion

The enormous intestinal mucosa is equipped with important oxidative and conjugated capacity in the first-pass metabolism. Detoxification is achieved through differential expression of a wide range of detoxifying and drug metabolizing enzymes, such as cytochrome P450 and UGT enzymes. Using DRT-PCR, UGT1A regulation was analyzed in fifty cancerous tissue samples and their surrounding healthy tissue samples, forty normal colonic tissue samples and twenty four normal liver tissue samples from healthy controls. We observed that the control of UGT1A mRNA expression is specific from tissue to tissue, level of specific tissue expression in order of relative coefficient are hepatic tissue, normal colonic tissue, surrounding healthy tissue and colonic cancerous tissue (Fig.1,2,Table.1,2). Moreover, colonic UGTlA gene expressions were characterized by significant individual variation, whereas hepatic UGTlA mRNA expressions were more homogeneous. To the best of the authors’ knowledge, presently, this is the only study to document the down-regulation of UGT1A mRNA in colonic cancerous tissue and its surrounding healthy tissue. To further test our hypothesis, we tested the transcriptional regulation of UGTlA isoforms in the liver and three extrahepatic tissues. A pattern of tissue-specific gene expression was observed. Colonic cancerous tissues exhibited considerable differences from that found in the liver and normal colon tissues (Fig.1).

Importantly, expression of UGT1A isoforms showed no significant variation between individual hepatic samples, a finding that reflects the absence of polymorphic UGT transcript regulation in human liver. The colon tissue (UGT1A1, UGT1A3, UGT1A4, UGT1A6, UGT1A8, UGT1A9, and UGT1A10) have been characterized to express specific UGT1A transcript patterns with individual variation. Our finding suggests that, although liver express more abundant UGT1A, the greater diversity in UGT1A locus expression in human colon may reflect glucuronidation requirements necessary at the distal end of the gastrointestinal tract. Due to long exposure time of colon mucosa to intra-luminal compounds, and metabolism of mutagen-associated genotoxic and cytotoxic substances escaped from the proximal digestive system. The association of colorectal cancer (CRC) with UGT1A1*6 (G71R in exon-1) [30], and UGT1A7*3 had been reported in Caucasoid, with an OR of 2.75[38] ; African Americans and American white [37]; Chinese (Taiwan), with an OR of 2.75; and Chinese (mainland) with an OR of 4.9[30]; and Chinese, with OR of 1.59[39]. However, little is known of the correlation between UGT1A8 genotype and susceptibility of colorectal cancer for Chinese, which is the motivation behind this study. Based on our previous studies, polymorphic regulation of UGT1A exists at transcriptional and translation level[40]. The differences in UGT1A expression among colonic cancerous tissues, surrounding healthy tissues and normal colonic tissues is a sign of changes in catalytic activities of specific UGT1A as well as a decrease in glucuronidation in colonic mucosa, such decrease may contribute as a risk factor for colorectal cancer. Human UDP-glucuronosyltransferases are expressed in a tissue-specific fashion. A unique tissue specificity is maintained through the expression of UGT1A8, which may be considerably focused and specialized. It also had been reported that UGT1A8 possessed relatively broad catalytic ability toward both xenobiotic and endobiotic compounds[12], further confirmed the important role of UGT1A8 in vivo. We proposed that polymorphisms of UGT1A can serve as a marker for colorectal cancer. We undertook a case-control study in Shandong Province of China. UGT1A8 was cloned from RNA in blood samples and characterized by DNA sequence analysis. Three SNPs (bp518 (CG), bp765 (AG), and bp830 (GA)) were found in the 1033bp fragment of UGT1A8 exon-1. Two of the SNPs are missense mutation at codons 173, and 277, where alanine, cysteine is replaced with glycine, tyrosine, respectively. It is previously established that the amino acid sequences of UGT1A8 varies from case to case [12], [14], [27], [28], same was noted our results, because of mutations generated at T^4^A, T^40^A, F^121^S, K^133^R, A^154^G, A^173^G, T^202^A and M^212^L. Additional studies are needed to identify more variants of UGT1A8 in the human population and to determine whether structural variants relate to functional differences biochemically.

After analyzing the distribution of UGT1A8 alleles frequency, it was found that the wild type allele (UGTlA8*1) frequency was lower among cancerous group than control group. It indicated that UGTlA8*1 allele is a protective factor that reduction of relative risk of developing colorectal cancer. However, the frequency of mutational allele UGTlA8*3 was higher in cancerous group than control group (Fig.4), which established UGTlA8*3 allele as a risk factor for colorectal cancer. With further analysis of genotype, frequency of homozygous genotype UGTlA8*1 was noted to be much higher in control group than study group , while genotype UGTlA8*2/*3 and UGTlA8*1/*3 were genetic factors contribute to the increased susceptibility to colorectal cancer. Analysis of genotype gives additional evidence to our original conclusion: UGTlA8*3 allele is a risk factor for colorectal cancer independent of gender. Polymorphisms of UGTlA8 is a main risk factor of generation of colorectal cancer (P = 0.000), especially homozygous UGTlA8*3 genotype (P = 0.000). The mutation of UGT1A8 A ^173^G is a conservative change between two non-polar amine acid, but such change has not been found in other UGT1A isoforms. The conservation of amino acid at codon173 is important for the function of protein. But some scholars[27], [29] examined the ability of UGT1A8*1 and UGT1A8*2 to glucuronidate different substrates. It was found that both alleles have similar catalytic activity, and the replacement of alanine by glycine at codon 173 has little influence on the catalytic activity of the protein. These differences may result from methodological differences (stable cell lines VS. transient transfection), or structural differences between UGT1A8*1 and *2, such as T^40^A and G^154^A polymorphism[28] in the UGT1A8 cDNA.

In contrast, the mutation in UGTlA8*3 is of highly conserved single base pair at codon 277 leads to the substitution of tyrosine for cysteine, which deactivate UGT1A8 protein. Codon 277, located in UGT1A8 exon-1, plays a critical role in maintaining correct conformation of the protein. It is necessary for the formation of disulfide bridge with another cysteine, which is vital to the function of UGT1A8 protein by maintaining its tertiary structure. It had been observed that the missense mutation at codon 277, in region of UGT1A8 exon-1, resulted from the changes in amine acid sequence.

Accordingly, there is obvious difference between catalytic activities of wild type (UGT1A8*1) proteins and UGT1A8*3[27], [29]. The decrease in colonic glucuronidation causes imbalance in homeostasis. There is no significant difference in catalytic activity between predominant alleles: UGTlA8*l and UGTlA8*2. However, the nonfunctional allele UGTlA8*3, especially homozygous UGTlA8*3, is rare in healthy adults, which can be regarded as a meaningful marker in screening for colorectal cancer.

Expression of the individual UGTs isozymes in hepatic and gastrointestinal tissues could be differentially induced by dietary compounds. Thus, a limitation in our study was the retrospective assessment of diet. The expression level of individual UGTs isozymes were more or less effected by dietary habits and medicine (if any), resulting in biased ORs. But the bias due to disease-related changes in diet and medication were of small concern, because the patients were first diagnosed six months prior, without any therapeutic measure. Additionally, we didn't find significant differences between lifestyle of control group and study group before diagnosis. Another source of bias is the coexistence of haplotypes with different UGT1A locus variants and their linkage to polymorphisms in other members of the UGT1A subfamily. Studies have shown the association between UGT1A1*7 variant and promoter with the coding region SNPs of UGT1A1, UGT1A3, and UGT1A7[41]–[43].

Human UGT genes are precisely regulated by complex transcriptional and translational mechanisms[44], [45]. Furthermore, genetic and/or evolutionary differences among patient cohorts from different geographic origin may account for differences in UGT1A8 gene expression. Specimen sampling in different areas of the colon may also influence the detection of UGT1A8 mRNA. However, this study looked only at UGT1A8, not the other low frequency alleles present in the Chinese of Shandong province. One of advantage of this study is that all participants are from the same area with similar genetic background (Han Chinese). Another strength of the study is the large sample size with controls to evaluate the role of UGT1A8 polymorphism in colorectal cancer. The large sample size allowed us to eliminate different potential mutagens such as dietary and environmental mutagens as well as alcohol and tobacco induced mutagens. In addition, we observed significant linkage disequilibrium exists between the UGT1A8 polymorphisms. However, the effects of sampling error could not be ruled out, and the interaction between UGT1A isoforms and UGT1A8*3 as well as its effect on disease severity in patients is remain to be elucidated.

## Supporting Information

Table S1 **All primers for RT-PCR.**
(DOCX)

Table S2 **Polymorphic sites of UGTlA8 exon-1 at nucletides 518, 765 and 830.** There is on linkage between SNPs in UGTl A8*1 and other allales.
(DOCX)

Table S3 **Identity of amine acid between UGTlA isoforms and UGTlA8 mutational pattern (173 and 277).**
(DOCX)
