# Supplementary material for: Polymorphic Expression of UDP-Glucuronosyltransferase UGTlA Gene in Human Colorectal Cancer
Source: PLoS One. 2013 Feb 27;8(2):e57045. doi: 10.1371/journal.pone.0057045 (PMC3584141; doi:10.1371/journal.pone.0057045)
Supplement: Table S1 — All primers for RT-PCR. (DOCX) [file pone.0057045.s001.docx]

**Table.S1. All primers for RT-PCR.**

| Gene | Upstream primer | Downstream primer | Amplificatied segment (bp) |
| --- | --- | --- | --- |
| UGT1A | 5’-cgaatcttgcgaacaacacg-3’ | 5’-atgaaggccactgtcagcacg-3’ | 487 |
| UGT1A1 | 5’-aacaaggagctcatggcctcc-3’ | 5’-gttcgcaagattcgatggtcg-3’ | 644 |
| UGT1A3 | 5’-tgttgaacaatatgtct ttggtcta-3 ’ | 5’-ccaatgaagaccatgttgggc-3’ | 483 |
| UGT1A4: | 5’-gaaggaatttgatcgcgttac-3’ | 5’-ccaatgaagaccatgttgggc-3’ | 572 |
| UGT1A5 | 5’-ggtggtggtcctcaccctg-3’ | 5’-ccaatgaagaccatgttgggc-3’ | 659 |
| UGT1A6 | 5’-tcctggctgagtatttgggcc-3’ | 5’-gttcgcaagattcgatggtcg-3’ | 562 |
| UGT1A7 | 5’-tgccgatgctcgctggacg-3’ | 5’-gttcgcaagattcgatggtcg-3’ | 754 |
| UGT1A8 | 5’-ggtcttcgccaggggaatagg-3’ | 5’-gttcgcaagattcgatggtcg-3’ | 514 |
| UGT1A9 | 5’-ggaggaacatttattatgccaccg-3’ | 5’-gttcgcaagattcgatggtcg-3’ | 392 |
| UGT1A10 | 5’-cctctttcctatgt ccccaatga-3’ | 5’-gttcgcaagattcgatggtcg-3’ | 478 |
| β-actin | 5’-atcatgtttgagaccttcaaca-3’ | 5’-catctct tgctcga agtcca-3’ | 317 |
