# Supplementary material for: Polymorphic Expression of UDP-Glucuronosyltransferase UGTlA Gene in Human Colorectal Cancer
Source: PLoS One. 2013 Feb 27;8(2):e57045. doi: 10.1371/journal.pone.0057045 (PMC3584141; doi:10.1371/journal.pone.0057045)
Supplement: Table S2 — Polymorphic sites of UGTlA8 exon-1 at nucletides 518, 765 and 830. There is on linkage between SNPs in UGTl A8*1 and other allales. (DOCX) [file pone.0057045.s002.docx]

**Table.S2. Polymorphic sites of UGTlA8 exon-1 at nucletides 518, 765 and 830. There is on linkage between SNPs in UGTl A8*1 and other allales.**

|  | Nucleotide sites | | |
| --- | --- | --- | --- |
|  | 518 | 765 | 830 |
| UGT1A8*1  varients | C | A | G |
|  | G | G | A A |
|  | A173G | T255(silent) | C277Y |
