# Supplementary material for: Polymorphic Expression of UDP-Glucuronosyltransferase UGTlA Gene in Human Colorectal Cancer
Source: PLoS One. 2013 Feb 27;8(2):e57045. doi: 10.1371/journal.pone.0057045 (PMC3584141; doi:10.1371/journal.pone.0057045)
Supplement: Table S3 — Identity of amine acid between UGTlA isoforms and UGTlA8 mutational pattern (173 and 277). (DOCX) [file pone.0057045.s003.docx]

**Table.S3.Identity of amine acid between UGTlA isoforms and UGTlA8 mutational pattern (173 and 277).**

| nucleotide sites | | | | | | |
| --- | --- | --- | --- | --- | --- | --- |
| UGT | 172 | 173 | 174 | 276 | 277 | 278 |
| 1A1 | L | P | C | N | C | L |
| 1A3 | I | P | C | N | C | A |
| 1A4 | I | P | C | N | C | A |
| 1A6 | F | P | C | N | C | K |
| 1A7 | I | F | C | N | C | H |
| 1A8*1 | I | A | C | N | C | H |
| 1A8*2 | I | G | C | N | C | H |
| 1A8*3 | I | A | C | N | Y | H |
| 1A9 | I | L | C | N | C | H |
| 1A10 | I | F | C | N | C |  |
